# Midesophageal diverticulum with elevated intrabolus pressure: a case report

**DOI:** 10.1186/s40792-024-01909-7

**Published:** 2024-05-03

**Authors:** Kaito Mihara, Shigeru Tsunoda, Tatsuto Nishigori, Shigeo Hisamori, Shintaro Okumura, Keiko Kasahara, Yusuke Fujita, Takashi Sakamoto, Tomoki Morimoto, Hiromitsu Kinoshita, Yoshiro Itatani, Nobuaki Hoshino, Ryosuke Okamura, Hisatsugu Maekawa, Koya Hida, Kazutaka Obama

**Affiliations:** Department of Surgery, Graduate School of Medicine, Kyoto University, 54 Kawahara-Cho, Shogoin, Sakyo-Ku, Kyoto, 606-8507 Japan

**Keywords:** Diverticulum, Dysphagia, Esophagus, Myotomy, Thoracoscopy

## Abstract

**Background:**

Esophageal diverticulum is commonly associated with esophageal motility disorders, which can be diagnosed using high-resolution manometry (HRM) according to the Chicago classification. Although midesophageal diverticulum (M-ED) is associated with inflammatory processes, esophageal motility disorders have been recently identified as an etiology of M-ED.

**Case presentation:**

We present the case of a patient with M-ED and elevated intrabolus pressure (IBP), which did not meet the criteria for esophageal motility disorders according to the Chicago classification. A 71-year-old man presented with gradually worsening dysphagia for two years and was diagnosed as having an 8-cm-long M-ED and multiple small diverticula in lower esophagus. HRM revealed a median integrated relaxation pressure of 14.6 mmHg, a distal latency of 6.4 s, and an average maximum IBP of 35.7 mmHg. He underwent thoracoscopic resection of the M-ED and myotomy, which successfully alleviated the symptoms and reduced the intrabolus pressure to normal levels.

**Conclusions:**

It is important to recognize the esophageal diverticulum pathology with HRM findings even in cases where the results may not meet the Chicago classification and to include myotomy based on the results.

## Background

Esophageal diverticulum (ED) is a rare disease, with midesophageal diverticulum (M-ED) accounting for 15% of all ED cases [1]. Although most patients with ED are asymptomatic, surgery is indicated in symptomatic cases. Symptoms of ED vary and include dysphagia, chest pain, regurgitation, respiratory complications, and weight loss [2, 3]. Epiphrenic esophageal diverticulum (E-ED) is associated with esophageal motility disorders. While the development of M-ED is related to inflammatory changes due to tuberculosis or other etiologies, recent studies report esophageal motility disorders as a cause of M-ED as well [4].

Esophageal motility disorders are categorized by the Chicago Classification using metrics from high-resolution manometry (HRM). Intrabolus pressure (IBP) is one of the HRM metrics, and the measurement of the compartmentalized pressure exerted on a solid or liquid bolus transiting through the esophagus, Although IBP has been suggested to be associated with esophageal motility disorders [5], there are still no reports of esophageal diverticulosis associated with IBP.

Here, we report the first case of a patient with M-ED in whom elevated IBP suggested an esophageal motility disorder, who symptomatically improved following thoracoscopic diverticulectomy and myotomy with declined IBP.

## Case presentation

**Fig. 1:**
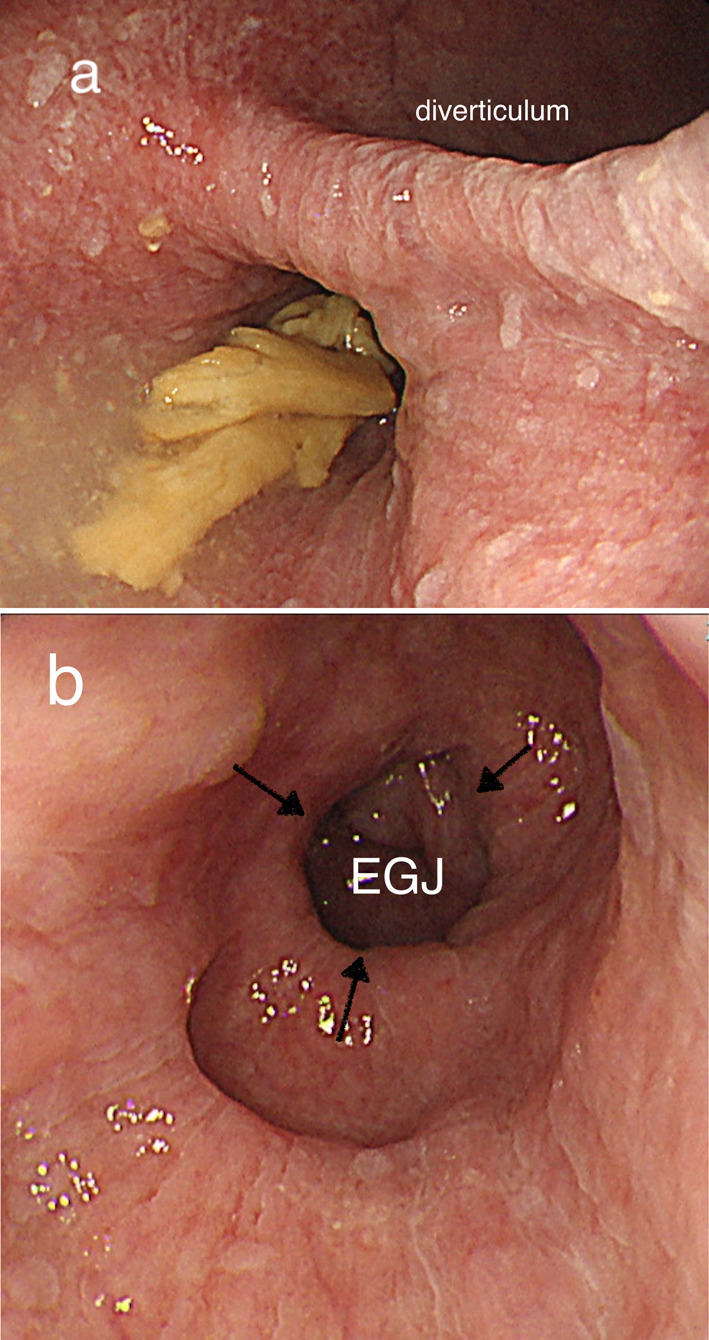
Preoperative esophagogastroduodenoscopy. **a** Large esophageal diverticulum. **b** Stenotic area (arrow)

A 71-year-old man presented to a regional hospital with gradually worsening dysphagia for two years. He had not experienced any dysphagia or vomiting before, nor had he ever undertaken an endoscopy. Esophagogastroduodenoscopy revealed a giant M-ED and multiple small diverticula in lower esophagus with mild stenosis, although the endoscope passed through it without difficulty (Fig. 1). He was referred to our hospital for surgery.

**Fig. 2:**
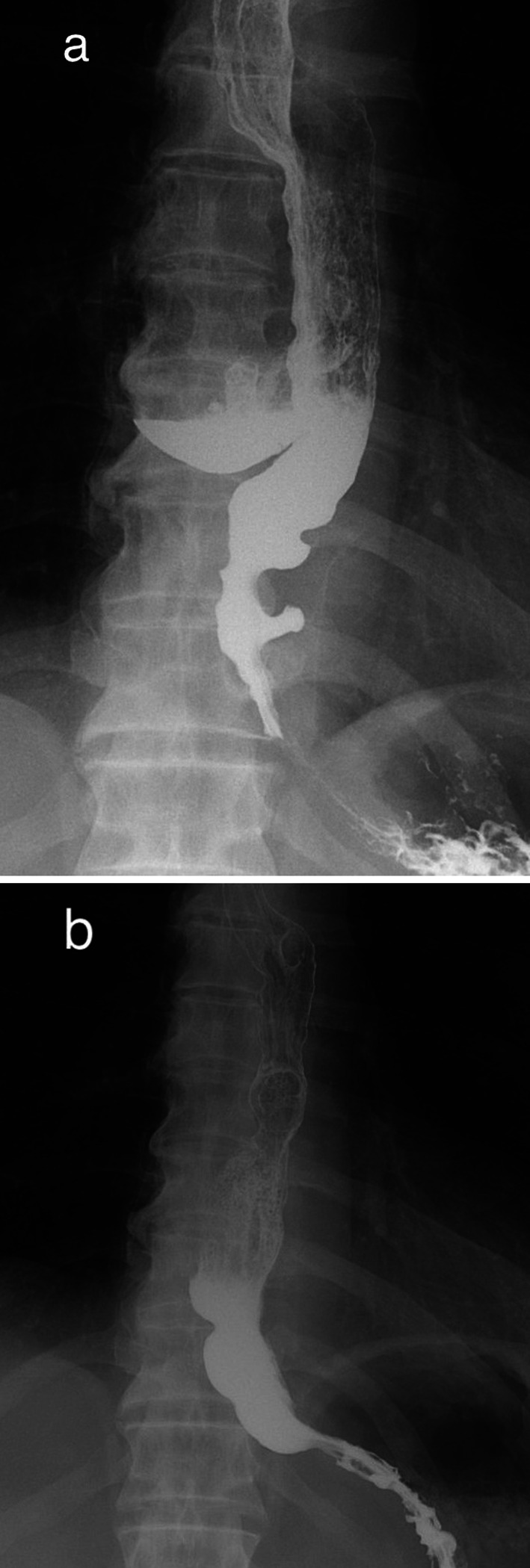
Barium swallow test. **a** Prior to surgery, barium swallow test shows a diverticulum on the right wall and several small diverticula in lower esophagus. **b** Postoperative barium swallow test reveals the disappearance of all diverticula

**Fig. 3:**
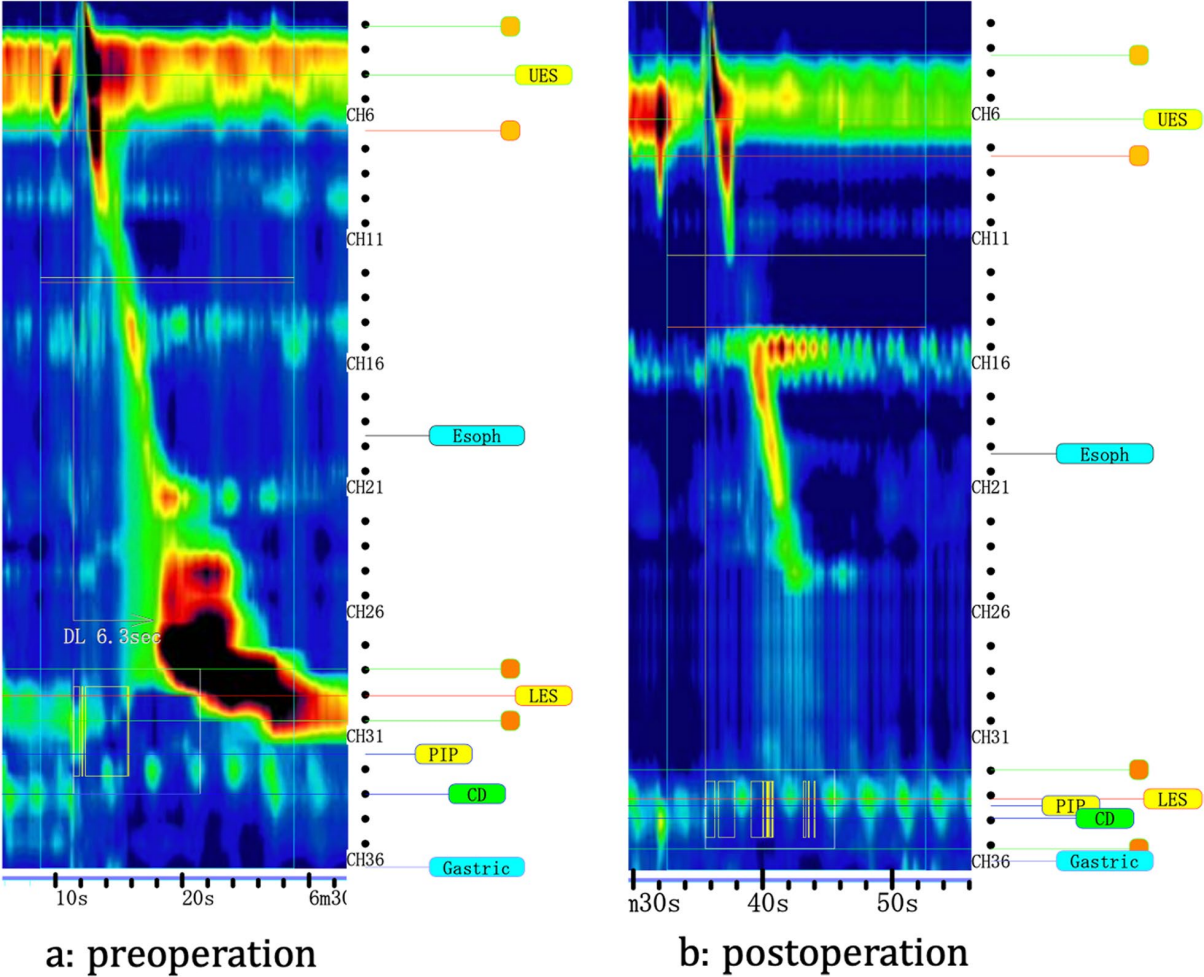
High-resolution manometry test. **a** Prior to surgery, intra-esophageal pressure in the lower esophagus tended to be high. **b** Intra-esophageal pressure has normalized after surgery

Computed tomography revealed an 8-cm-long M-ED on the right side of the esophagus. Barium swallow test confirmed the diagnosis of M-ED and multiple small diverticula in lower esophagus with some narrowing though barium promptly passed through the esophagogastric junction (EGJ) (Fig. 2a). HRM (Starlet, Star Medical, Inc, Tokyo, Japan) revealed a median integrated relaxation pressure (IRP) of 14.6 mmHg (upper cutoff; 26 mmHg), a distal latency (DL) of 6.4 s (lower cutoff; 4.5 s), and an average maximum IBP of 35.7 mmHg (Fig. 3a). The patient did not meet the criteria for an esophageal motility disorder according to the Chicago classification (version 4.0) [6]; however, elevated IBP (> 20.1 mmHg) suggested the presence of an underlying structural restriction or obstruction at the EGJ [7].

Thoracoscopic resection of the M-ED and myotomy of the lower esophagus were performed. Briefly, the patient was placed in a prone position with bilateral lung ventilation and approached from the right chest under artificial pneumothorax at 10 mmHg with carbon dioxide. A 12-mm camera port and three working ports were inserted at the 5th, 7th, and 9th intercostal spaces. The diverticulum in mid esophagus was identified through the pleura as a pulsion diverticulum without muscularis propria, and the right vagus nerve was running near the ED. The root of the ED was exposed preserving the right vagus nerve and longitudinally transected using linear staplers, and the muscle layers were sutured with 3–0 proline to bury the staple lines. Myotomy was performed in the right posterior wall of the esophagus, extending from immediately below the ED to the level of the diaphragm, ~ 12 cm in length. The lower esophagus looks normal in appearance, but the muscular layer was thickened just as same as achalasia during the myotomy. Finally, intraoperative endoscopy confirmed sufficient opening of the midthoracic esophagus and improvement of the stenosis in the lower esophagus. The operation duration was 119 min, and intraoperative blood loss was minimal.

Histopathologic investigation revealed that M-ED was composed of mucosa and submucosa without the muscle layer and was diagnosed as pseudodiverticulum. Postoperative esophagography showed smooth passage of the contrast medium without leakage or stenosis (Fig. 2b). The postoperative course was uneventful, and the patient was discharged on postoperative day 13. His dysphagia had completely resolved at the 3-month follow-up visit, and the HRM test performed at that time demonstrated that the average maximum IBP was declined to within the normal range (Fig. 3b).

## Discussion

EDs are classified based on their anatomy (true or false) and etiology (traction or pulsion) [4]. E-EDs are often false pulsion diverticula secondary to an esophageal motility disorder [8]. Conversely, M-EDs are usually true traction diverticula that are secondary to postinflammatory scarring and are classically called Rokitansky diverticula [4]. However, our patient did not exhibit periesophageal inflammation, and the histopathology confirmed the diagnosis of a pseudodiverticulum. These findings resembling E-ED suggest a similar etiology at play. Esophageal motility disorders, which are associated with 43–100% of all E-ED cases [9, 10], are also associated with M-ED [3, 11, 12]. HRM metrics are essential for classifying esophageal motility disorders. The IRP is a measure of deglutitive relaxation based on 4 s of the lowest mean axial pressure, continuous or discontinuous, across the LES during the 10-s period after a swallow, and is an important metric to assess adequacy of EGJ relaxation. The DL is a time measurement from the start of swallow-induced UES opening to the arrival of esophageal contraction at the contractile deceleration point, the inflection point in the wavefront velocity proximal to the EGJ. A swallow is considered premature or spastic if the DL is less than 4.5 s. For example, one of the esophageal motility disorders, Type I achalasia, is diagnosed by the absence of normal esophageal peristalsis and IRP > 25 mmHg with at least 2 swallows. In the present case, the IRP was normal, and the DL was normal. Therefore, the patient did not meet the criteria for an esophageal motility disorder although the IBP was elevated, which can indirectly indicate esophageal obstruction and is associated with esophageal motility disorders [5, 13]. The fourth version of the Chicago classification has adopted IBP as a criterion for ED, although IBP is recommended for the evaluation of motility disorders only in patients with an abnormal IRP [6]. Quader et al. reported that elevated IBP indicated an obstructive process in cases where IRP is normal [7]. In the present case, the elevated IBP might be due to an obstructive issue, although the IRP was normal. This is the first case reporting a patient with M-ED in whom elevated IBP suggested an esophageal motility disorder.

The indication for surgery should be carefully considered in patients with M-ED. Those with asymptomatic ED are often followed without surgery as they do not experience clinically significant progression of symptoms [14]. On the other hand, symptoms such as dysphagia and pneumonia can lead to reduced quality of life and sometimes fatal complications, leading to the consideration of surgery. However, postoperative complications, especially leakage, are not uncommon and require attention. In one study from Mayo Clinic on one of the largest cohort studies on open surgery for ED, mortality rate was 9.1% and 18% of the all patients experienced leakage [9]. Although minimally invasive surgery, including laparoscopic and thoracoscopic approaches, has become the mainstream in recent years, the overall mortality rate of 0–7% is up to 8–24% in those with leakage [4, 10, 12, 15]. Therefore, surgery for ED is not a low-risk option even today despite significant surgical advances and indications for surgery should be carefully considered.

Patients with M-ED and esophageal motility disorder should undergo esophagomyotomy and diverticulectomy, given that diverticulectomy alone is associated with an increased rate of esophageal leak and ED recurrence [16]. However, esophagomyotomy in patients without an esophageal motility disorder remains controversial; some surgeons argue that myotomy should not be added to the surgical approach as it may increase the risk of leakage from unnecessary myotomy [3, 12], while others propose that myotomy should be performed in all cases because they consider that the patients have an underlying esophageal motility disorder not noted in the Chicago classification [10]. In the present case, although the patient did not meet the criteria for an esophageal motility disorder, elevated IBP indicated an esophageal motility disorder, and myotomy was performed in addition to diverticulectomy, leading to the successfully symptomatic resolution without recurrence.

## Conclusions

As the potential presence of a distal mechanical/functional obstruction of M-ED cannot be denied, the esophageal function should be thoroughly evaluated with the HRM test. Esophageal myotomy should be considered at the time of diverticulectomy in the presence of an esophageal motility disorder.

## Data Availability

Data are available upon reasonable request from the corresponding author.

## Abbreviations

ED: Esophageal diverticulum
M-ED: Midesophageal diverticulum
E-ED: Epiphrenic esophageal diverticulum
EGJ: Esophageal junction
HRM: High-resolution manometry
IBP: Intrabolus pressure
IRP: Integrated relaxation pressure
DL: Distal latency
